# The Rich World of p53 DNA Binding Targets: The Role of DNA Structure

**DOI:** 10.3390/ijms20225605

**Published:** 2019-11-09

**Authors:** Václav Brázda, Miroslav Fojta

**Affiliations:** Institute of Biophysics of the Czech Academy of Sciences, Královopolská 135, 612 65 Brno, Czech Republic; fojta@ibp.cz

**Keywords:** p53, protein-DNA interactions, consensus sequence, cruciform, local DNA structures

## Abstract

The tumor suppressor functions of p53 and its roles in regulating the cell cycle, apoptosis, senescence, and metabolism are accomplished mainly by its interactions with DNA. p53 works as a transcription factor for a significant number of genes. Most p53 target genes contain so-called p53 response elements in their promoters, consisting of 20 bp long canonical consensus sequences. Compared to other transcription factors, which usually bind to one concrete and clearly defined DNA target, the p53 consensus sequence is not strict, but contains two repeats of a 5′RRRCWWGYYY3′ sequence; therefore it varies remarkably among target genes. Moreover, p53 binds also to DNA fragments that at least partially and often completely lack this consensus sequence. p53 also binds with high affinity to a variety of non-B DNA structures including Holliday junctions, cruciform structures, quadruplex DNA, triplex DNA, DNA loops, bulged DNA, and hemicatenane DNA. In this review, we summarize information of the interactions of p53 with various DNA targets and discuss the functional consequences of the rich world of p53 DNA binding targets for its complex regulatory functions.

## 1. Introduction

p53 is the most often mutated tumor suppressor in humans and is studied intensively from different points of view due to its crucial role in malignant transformation [1,2,3]. p53 functions include regulatory roles in processes such as ontogenesis [4,5,6], myogenesis [7], metabolism [8], cell cycle arrest [9,10], apoptosis [11,12], angiogenesis [13,14], DNA repair [15,16], and cell senescence [16,17]. These numerous roles are realized through its interactions with other proteins and DNA. Although p53 mediated gene transcription is mostly connected to its direct interaction with DNA, it has also been shown that p53 interacts with many proteins, including several transcription factors and regulators [18]. Especially for mutant p53, its transcriptional functions relate to its ability to interact with other transcription factors [19]. Both activation, for NF-Y [20], or repression, for p63 and p73 [21,22], has been demonstrated. However, many other transcription factors interact with p53 [23]. p53 plays roles in multiple pathways [24,25,26] by acting as a transcription factor, therefore requiring DNA binding activity [27,28,29]. Compared to many other transcription factors, p53 DNA targets are not defined by a particular consensus sequence, but p53 is able to bind various DNA sequences and DNA targets defined by their secondary structures. Many of these local DNA structures are conserved throughout evolution and play essential roles in regulating many biological processes [30,31,32]. There are several types of these “alternative” DNA structures such as cruciforms, left-handed DNA (Z-DNA), triplexes and quadruplexes [33,34]. Bioinformatic analyses demonstrated non-random locations within the genome for certain local DNA structures, for example origins of replication (cruciforms), promoter regions (cruciforms, triplexes, and G-quadruplexes), introns (triplexes) and telomeres (G-quadruplexes) [35,36,37,38,39,40,41]. In keeping with their important regulatory roles, many local DNA structures are important also in human diseases, for example triplex structure in the frataxin gene caused by GAA/TTC triplet expansion is associated with Friedreich’s ataxia [42,43]. Many studies have also shown dysregulation of local DNA structures in cancer cells and especially G-quadruplexes are being tested as a target for cancer treatment [44,45]. The interactions of p53 with DNA target sequences have recently been reviewed in several papers [2,46,47,48,49,50,51,52], including detailed analyses of the p53 family consensus sites and its non-canonical sequences with different lengths, variations of the core and flanking sequences and spacers (see below) [53]. Therefore, in this review, we focus on p53 binding properties to DNA targets in non-B-DNA conformation and to local DNA structures. We summarize published data about these interesting p53 DNA binding properties and hypothesize their possible roles.

## 2. Various DNA Targets of p53

### 2.1. ChIP-Seq and p53-Target Sequences

DNA sequence-specific binding of p53 was initially determined by the SELEX method and has been verified by many approaches in vitro and in vivo [54,55]. Complete mutagenesis of the p53 DNA target, which is typically formed by two copies of a 5′-RRRCWWGYYY-3′ sequence (where R represents purine, Y represents pyrimidine and W represents adenine or thymine bases), provides information concerning p53 binding affinities for all possible consensus p53 targets and it is easy to compute the theoretical p53 binding affinity to the target in its linear B-DNA form [56,57]. p53 is also able to search for its target by sliding [29,58,59] and can promote intersegmental transfer by binding to two DNA strands simultaneously [60]. Contemporary analyses of datasets from genome-wide chromatin immunoprecipitation (ChIP) of p53-bound DNA fragments followed by high-throughput sequencing has led to the development of p53 BAER (a human p53 Binding And Expression Resource) [61] that can be accessed on the University of California Santa Cruz (UCSC) Genome Browser [62,63]. These complex analyses confirmed and validated some known data about p53 sequence-specific DNA binding and p53 consensus sequence(s), showing, for example, that the most common is the consensus sequence without spacers and that many precipitated fragments contain only half of the consensus sequence [61]. In addition, these analyses brought many new and unexpected results. For example, only 35% of the sequences are in the range of 5 kbp before the transcription start site (TSS), while 25% are found in intragenic and 41% in intergenic regions. Assuming that the main role of p53 is associated with transcription, it could be expected that the majority of p53-target sequences will be found in promoter regions. Therefore, the above-mentioned results provoke the question: what is the role of p53 binding to regions which are far away from TSS and therefore could hardly regulate transcription? 

Based on homology with the consensus binding site or the presence of half-binding sites, it is possible to predict potential p53 binding sites in the human genome and their theoretical affinities and several tools are available for these purposes [56,57,64]. Using this approach, almost 800,000 potential p53 target sequences containing a p53-like motif consisting of the p53 consensus 20-mer with a 0–15 bp spacer or with only a half-site have been identified in the human genome [48]. However, data from 41 ChIP datasets identify only 54,947 p53-bound sequences, of which just 12,885 (23%) contain either a full or half-site p53 consensus sequence [61]. Thus, from all of the predicted potential p53 targets in the human genome (almost 800,000), only ∼1.6% are actually bound by p53 and most p53-bound DNA fragments (77%) do not contain a consensus binding site. From these analyses, it is clear that multiple factors are required to enable p53 binding to DNA. Moreover, what does p53 protein recognize in the genome when the classic p53 double-stranded B-DNA target sequence is not present? Interestingly, a substantial correlation exists amongst the p53 ChIP-seq data and the presence of CpG islands [61,65]. Moreover, many studies have now demonstrated that p53 binds to various non-B DNA targets, as reviewed below.

### 2.2. p53 Binding to Distorted Double-Stranded DNA

**Figure 1:**
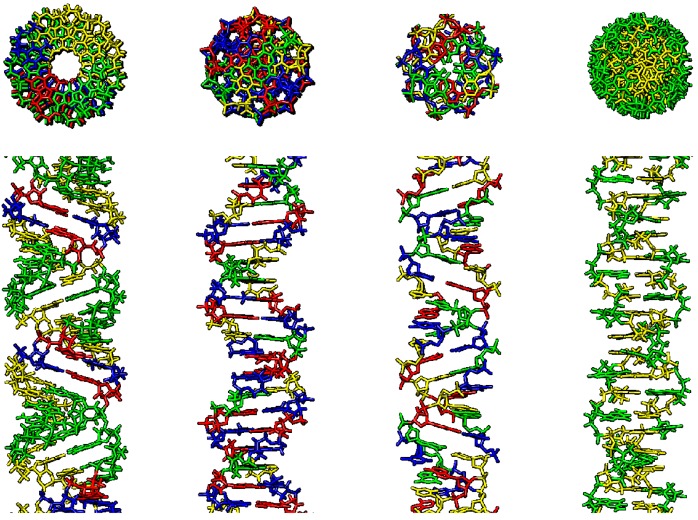
Various double-stranded DNA conformations, right-handed: A-DNA, B-DNA, C-DNA, left-handed: Z-DNA (from left to right). First row–upper view, second row–side view. A-DNA–AGGGGCCCCT repeat, B-DNA–random sequence, Z-DNA–CG repeat. Visualized using Chimera software, A–blue, C–yellow, G–green, T–red.

The majority of DNA exists in the double-stranded form in the genomes of both prokaryotic and eukaryotic organisms. However, DNA is a structurally flexible molecule and exists also in single-stranded, three-stranded and four-stranded variations. Most in vitro p53-DNA affinity studies have been performed with short double-stranded oligonucleotides containing a p53 consensus sequence. DNA in the cell nucleus is not present in such a form: nuclear DNA is involved in long linear chromosomal structures and is organized on several levels. Thus, accessibility of genomic DNA is dependent on its organization and interaction with diverse proteins. It is an inherently complex process to pack long chromosomes into a structure that fits into the nucleus. Moreover, this structure must not be rigid but has to be flexible to allow functional roles of DNA. Therefore, DNA in the nucleus can be present as double-stranded DNA not only in the “standard” B-DNA conformation but also in different double-stranded variations such as A-DNA, C-DNA, and Z-DNA (Figure 1). Many studies have shown that p53 binds to damaged, mismatched, and/or distorted double-stranded DNA [66,67,68]. These p53 binding modes are crucial for p53 stabilization and trigger its posttranslational modification and accumulation, leading to its sequence-specific binding. From this point of view, DNA distortions and/or transitions between various double-stranded DNA conformations may be involved in rapid p53 activation for sequence-specific DNA binding (particularly when sites of structural variation are located in close proximity to the p53 sequence-specific motif).

**Figure 2:**
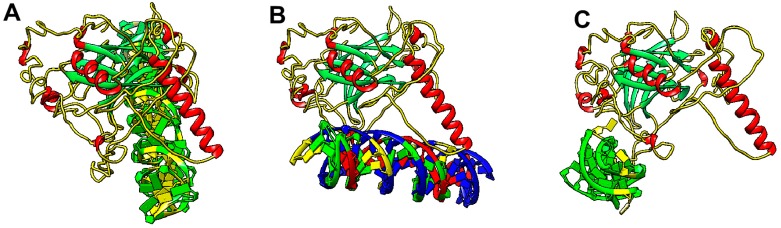
In silico model of human p53 interactions with Z-DNA (**A**), Triplex DNA (**B**) and G-quadruplex DNA (**C**). Visualized using Chimera software, Protein: helices-red, beta-sheets-green, DNA: A–blue, C–yellow, G–green, T–red.

The p53 central domain is the major region responsible for sequence-specific binding to double-stranded B-DNA and this region contains all of the common, hotspot, mutations seen in cancer. These hotspot mutations either alter the conformation of the DNA binding domain (such as R175H) or involve mutation of an amino acid that directly contacts DNA (such as R273H), reducing or abolishing sequence-specific transactivation [69,70,71]. The crystal structure of the p53 core domain with a linear DNA target has shown the importance of individual amino acid residues interacting with DNA [72] and were verified several times [73,74,75,76]. It has been also demonstrated that DNA bending is important for p53 target sequence recognition [77]. Due to the lack of data for full-length p53 protein interactions with non-B DNAs, we used PDB data of p53 structure (combined with de novo predictions of particular unresolved regions by i-TASSER [78]) and Z-DNA, Triplex and G-quadruplex DNA PDBs to predict p53 interactions with these structures in silico by HDOCK tool [79]. The results of these analyses were visualized in UCSF Chimera software [80] and are shown in Figure 2. Various parts of the protein are predicted to interact with various DNA structures. While Z-DNA interacts with p53 mainly at residues in the central part (Figure 2A), triplex (Figure 2B) and G-quadruplex (Figure 2C) interacting residues are located mainly in the C-terminal domain, which corresponds to the experimental observations discussed below.

### 2.3. p53 Binding to DNA Structures Presented in Single-, Triple- and Four-Stranded DNA Motifs

**Figure 3:**
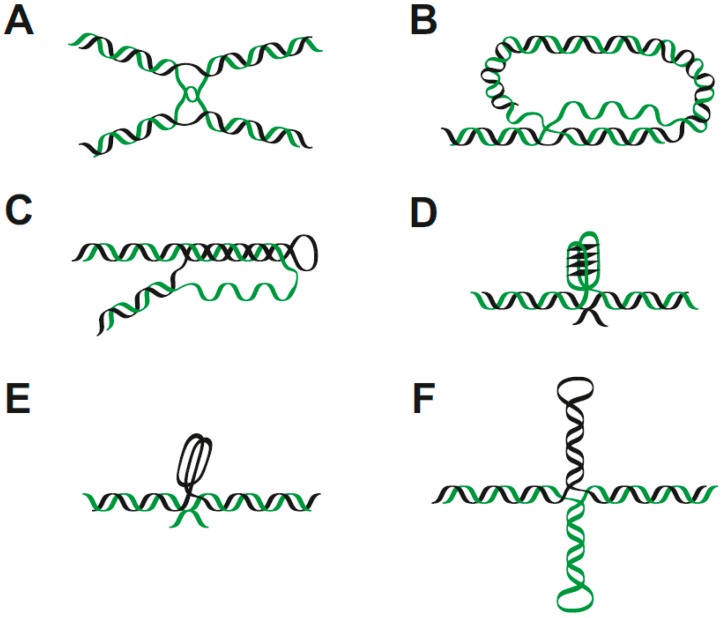
DNA structures with various numbers of strands (**A**) hemicatenate DNA (single- and four-stranded parts), (**B**) T-loop (single-, four-stranded part with DNA junction) (**C**) Triplex DNA (single- and three-stranded DNA), (**D**) G-quadruplex (four-stranded) (**E**) I-motif (four-stranded) (**F**) Cruciform with loops (single-, double- and four-stranded junction). Parts B, C, D, and F are adapted from [83].

Besides the variations in double-stranded DNA structures, important regulatory roles have been ascribed to secondary DNA structures consisting of different numbers of chains and/or involving single-stranded parts. The classic example of secondary structure in nucleic acids is the presentation of RNAs as folded single-stranded molecules forming various functional three-dimensional structures with or without protein parts, such as tRNAs, ribosomal RNAs, SRP RNA, snRNA, snoRNA, lncRNA, etc. However, several alternative DNA structures have been characterized in vitro and their existence has recently been demonstrated in vivo. Considering that the preferential binding of p53 to single-stranded DNA was shown more than thirty years ago [81,82], it is perhaps not surprising that p53 binding to several local DNA structures present as non-B (or more generally non-double-stranded) DNA was described. The overview of various local DNA structures with different numbers of DNA strands is shown in Figure 3.

Besides the recent ChIP-seq results showing that a remarkable number of precipitated DNAs do not contain the p53 consensus sequence, results showing preferential binding of wild-type p53 to supercoiled DNA (both containing and lacking the consensus sequence) pointed to p53 structure-specific DNA binding [84,85,86,87]. Selective binding to supercoiled DNA was later demonstrated for mutant p53 (tested for seven hot spot mutant p53 proteins (R175H, G245S, R248W, R249S, R273C, R273H and R282W), while the same plasmid DNAs in linear or relaxed circular forms were poorly bound in the absence of a consensus sequence [88]. This preference for supercoiled DNA has also been confirmed in cells using ChIP. These studies were initially performed on a circular plasmid with negative supercoiled DNA and later also for positively supercoiled DNA [89,90]. Experiments with superhelical DNA topoisomers revealed that p53 prefers DNA molecules with higher numbers of super turns [90,91,92]. Notably, negative DNA superhelicity is known to stabilize different non-B DNA structures. An increasing number of papers are demonstrating the regulatory importance of these local DNA structures in the human genome [93,94,95] and, for example, G-quadruplexes have been suggested as promising targets in cancer treatment [44,45,96]. Interestingly, inhibition of topoisomerases, resulting in accumulation of DNA superhelicity, leads to changes in p53-directed regulations in human cell lines [97]. Considering the stabilizing effects of negative superhelicity on local DNA structures including cruciforms, triplexes, and quadruplexes [33,98], it seems that local DNA structure protrusions also play a role in the mechanisms behind p53 function. Indeed, p53 preferential interactions with various local DNA structures have been shown: p53 is able to bind to mismatched DNA duplexes [67], three-way or four-way junctions [99,100], telomere T-loops [101], hemicatenated DNA [102], DNA loops [43], cruciforms [103,104,105,106], triplexes [37], and quadruplexes [107,108].

#### 2.3.1. Hemicatenate DNA

Hemicatenates (Figure 3A) are essential intermediates of DNA replication, repair, and recombination [109]. These structures consist of distorted double helical, single-stranded and four-stranded motifs. Based on their length and base content, they can adopt various 3D structures, however, their basic characteristic—distortion of the B-DNA structure and shorter or longer single-stranded DNA chains—is typical for hemicatenanes. The presence of hemicatenate DNA can lead to DNA fragmentation and it is crucial to remove it before replication. Therefore, hemicatenanes are resolved by topoisomerases, and topoisomerase inhibition leads to increased strand breakage in DNA [110,111]. It has been demonstrated that p53 binds hemicatenate DNA in vitro by gel-shift assays [102,112]. Interestingly, not only one type of complex is formed by the interaction of p53 with hemicatenate DNA, but three different complexes can be formed, indicating that p53 can recognize both single-stranded loops present in the hemicatenate DNA and the central part of the structure which is multi-stranded. Whilst the p53 C-terminal domain has been proposed to play an important role in binding to supercoiled DNA, C-terminally deleted p53 still selectively interacts with hemicatenate DNA [102].

#### 2.3.2. Telomeric T-Loops

In contrast to circular genomes found in the majority of bacteria as well as mitochondria and plastids, the ends of linear chromosomal DNA form specific protective structures. For mammalian telomere formation, so-called T-loops are typical [113,114]. This T-loop structure (Figure 3B) is formed by a 3′ single-stranded overhang of at least one TTAGGG repeat, Holliday junction-like structure, distorted double-stranded DNA and C-rich and G-rich parts that are prone to form non-B DNA structures [115]. The p53 binding preference to Holliday junctions was shown more than twenty years ago [99]. Therefore, p53 binding to T-loops is not surprising and p53 interacts with T-loop structures as a tetramer or as two tetramers [101]. Furthermore, p53 binds single-stranded TTAGGG with high affinity in oligonucleotides as well as in the presumably double-stranded form in plasmid DNA [101]. Moreover, the strand transfer activity of p53 may involve the formation of T-loops and cooperatively support TRF-2-mediated formation of the T-loop structure in vivo [115]. Both of these proteins have been detected together at the T-loop junction, suggesting the importance of p53 in T-loop formation and/or maintenance [101]. On the other hand, direct interactions of p53 with T-loop DNA have not been proven in vivo and it seems that DNA damage at human telomeres is prevented by p53 indirectly [116,117]. 

#### 2.3.3. Three-Stranded Structure

Repetitive sequences with mirror symmetry, consisting of homopurine homopyrimidine tracks, are capable of forming so-called triplex structures (Figure 3C), involving a segment of DNA with three nucleic acid strands. The third strand is typically bound by Hoogsteen pairing in the deep groove of the double-stranded DNA. Intramolecular triplexes are formed via the refolding of a segment of duplex DNA, which results in leaving a part of the DNA single-stranded. The formation of a triplex structure leads to torsions in surrounding parts of the DNA molecule [118]. Intermolecular triplexes with either third DNA or RNA strands have been described [119]. Triplex-forming sequences occur non-randomly in the human genome and have been found by bioinformatic approaches in several gene promoters, for example the *IL2R*, *POLA1*, and *MYC* genes [120], implying that triplex structures are involved in transcriptional regulation. In this regard, it has been demonstrated recently that formation of RNA-DNA triplexes leads to transcriptional inhibition in human cell lines. Formation of these RNA-DNA triplexes by interactions of long non-coding RNA with DNA could, therefore, be involved in additional or alternative transcription regulatory mechanisms. p53 binding to a plasmid DNA with confirmed presence of a triplex structure shows an increased affinity in comparison with supercoiled DNA without the triplex-forming DNA sequence [37]. Interestingly, this preferential binding to triplex-containing DNA was reduced by pre-treatment with monoclonal antibodies that bind to and block the C-terminal domain of p53. This result is in agreement with the in silico model of p53-triplex interaction (Figure 3B). Compared to p53 binding to hemicatenate DNA, it seems that the C-terminal domain of p53 plays a crucial role in triplex recognition. On the other hand, in vitro ELISA showed that both the core and the C-terminal p53 domains are capable of binding TAT triplex. Thus, cooperation of these two domains of p53 seems to be important for recognition of triplex structures [37]. Very interesting results were shown by luciferase reporter assays and RT-PCR: while an isolated triplex structure in a plasmid introduced into a human cell line showed no influence on transactivation by p53, significant enhancement of p53 dependent transactivation was detected when the same triplex-forming sequence was present next to the p53 consensus target sequence [37]. 

#### 2.3.4. Four-Stranded Structures

G/C-rich nucleic acid sequences are prone to form two types of quadruplex structures: G-quadruplexes (Figure 3D) formed by G tetrads, or i-motifs (Figure 3E) formed by two intertwisted C-loops [121,122]. To date, the majority of research has been focused on G-quadruplexes, which can form thermodynamically more stable structures in physiological conditions compared to double-stranded B-DNA with the same sequence [123,124]. The arrangement of G-quadruplexes varies depending on the G-track repetition length, number and kind of bases interrupting the G-tracks, ionic conditions, etc., as reported in several studies [30,125,126]. Nevertheless, the principal structural features are common for all G-quadruplexes: the arrangement of guanine quartets stabilized by Hoogsteen hydrogen interaction, the presence of a (usually) monovalent ion (optimally K^+^) in the middle of the G-quad bucket, single-stranded loops at the top and bottom parts of the structure and a four-way junction where the G-quadruplex structure is attached to the double-stranded DNA. Current research has emphasized the significance of G-quadruplexes in numerous cellular processes such as DNA replication, telomere maintenance and the binding and activity of transcription factors [127,128,129]. Hot-spot mutant p53 proteins bind weakly or do not bind to p53 target sequences [2,130] and these weak p53-DNA interactions are insufficient for effective transcription activation [131,132,133,134]. On the other hand, both wild type and mutant p53 (R273H) are able to bind G-quadruplexes. The C-terminal domain is therefore suggested as more important for G-quadruplex DNA binding compared to the central p53 domain, which is in agreement with the in silico model of p53-G-quadruplex interaction (Figure 3C). Interestingly, p53-DNA binding affects transcription from G-rich regulatory regions [135]. Mutant p53 proteins are known to modify transcription levels via their interactions with intronic and intergenic sequences predisposed to form non-B DNA structures [136]. Enrichment of mutant p53 (R273H) bound to regions from 1 kb upstream to 1 kb downstream of TSSs overlaps with CpG islands and about 75% of mutant p53 binding regions are predicted to contain G-quadruplex motifs [135]. This suggests the ability of p53 to recognize quadruplex DNA structures in vivo. It was also shown that p53 stabilizes G-quadruplex structures, but the p53 interaction with the G-quadruplex could be also mediated by interaction with other G-quadruplex recognizing transcription factors, such as ETS1, SP1 and others [137,138,139]. p53 interacts with the G-quadruplex-forming sequence present in the *MYC* promoter and represses transcription in vitro and in human cell lines [107]. Interestingly, some quadruplex-stabilizing ligands, such as *N*-methyl mesoporphyrin IX, boost the interaction of p53 to the G-quadruplex formed by the human telomeric repeat sequence [108].

#### 2.3.5. Loops and Cruciforms

**Figure 4:**
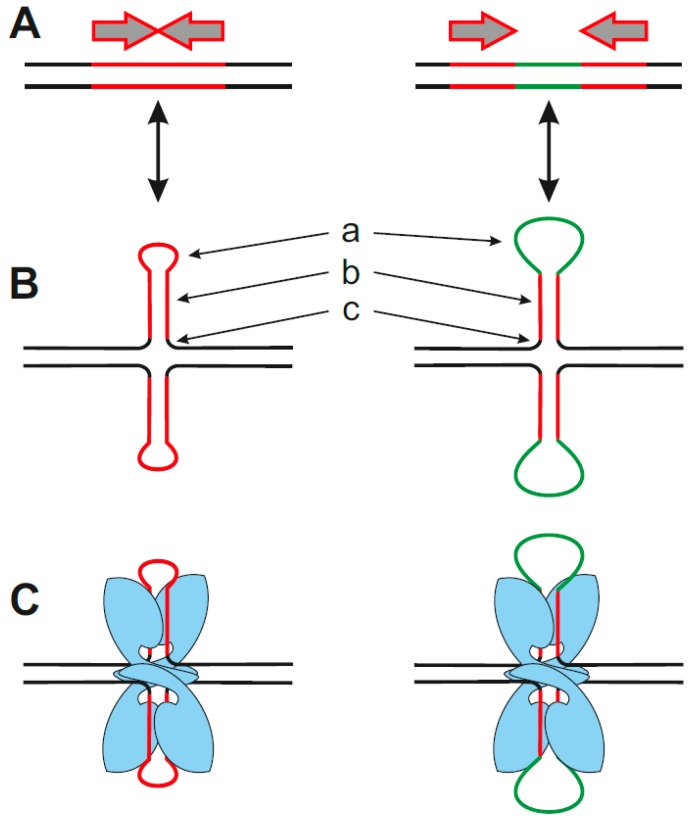
Cruciform structure formation (**A**) inverted repeat (red) without spacer (left part) or with spacer (green–right part) is required for (**B**) cruciform structure formation, the length of the repeat and spacer influence the length of the single-stranded loop (a), size of the stem (b). Every cruciform consist of four-way junction (c). Two p53 half-sites formed by inverted repeats could be close in cruciform structure allowing effective binding of p53 (blue) (**C**).

The presence of various repetitive sequences is typical for the genomes of all organisms. Many trinucleotide repeats have been described in the human genome, and their expansion is associated with neurological, degenerative and muscular diseases such as Friedreich’s Ataxia and Huntington’s disease [42,140,141]. Genomic elements with trinucleotide repeats are very flexible and are able to form non-B DNA structures such as loops, hairpins, triplexes and slipped-strand structures [142,143]. An important regulatory role has been ascribed also to inverted repeats. Such palindromic sequences are often identified as protein targets [103,144,145]. They are prone to form cruciform structures, which consist of a branch point (represented by a four-way junction), a stem (double-stranded) and a loop (single-stranded). Depending on the length and on whether the inverted repeat is direct or separated by a spacer, cruciforms with shorter or longer single-stranded loops are formed (Figure 4).

**Figure 5:**
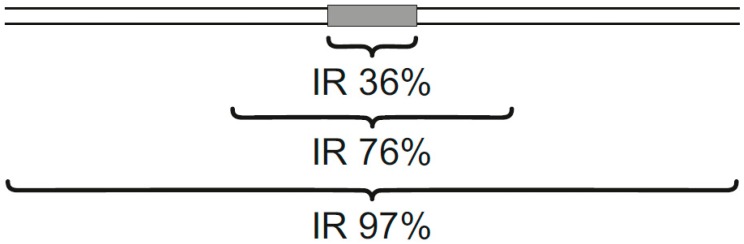
Frequencies and localization of inverted repeats in p53-ChIPed fragments, within a p53 double-stranded target, 20-bp around and 100-bp around.

Inverted repeats are present non-randomly in the genome and are often located in the proximity of breakpoint junctions, promoters, and replication origins [57,144,146]. Interestingly, many p53 target sequences in double-stranded DNA with a high transactivation activity in vivo contain inverted repeats [2,85,106] and there is a correlation between the presence of an inverted repeat in the p53 target site and enhancement of p53-DNA binding [92,147]. The formation of cruciforms within p53 double-stranded target sites facilitates p53-DNA binding in topologically constrained DNA [85]. A correlation between inverted repeat presence in the *CDKN1A* gene promoter and effective p53 binding was demonstrated by ChIP [104,148]. Analyses of genome-wide p53 ChIP data after cellular stress showed that the majority of sequences contain at least part of the p53 consensus sequence (93%) and 2245 of 2250 (99%) sequences contain at least one inverted repeat [52]. Moreover, inverted repeats are often present in close proximity (within 20 bp) of p53 target sequences (76% of p53-ChIPed sequences) and 34% contained the inverted repeat directly within the 20 bp long p53 consensus sequence (Figure 5). ChIP and yeast transactivation assays have demonstrated the preferential binding of p53 to cruciform-forming inverted repeats within p53 target sequences [104,106]. Most probably, better accessibility and stability of the protein-DNA complex leads to the improved p53 function at the fully symmetrical p53 target sites. An enhancement effect of a DNA loop in close proximity to a p53 B-DNA target has also been shown for triplex DNA [37] and for DNA loops formed by triplex expansion associated with Friedreich’s ataxia [43]. The presence of single-stranded loops and/or four-way junctions in a cruciform could contribute to the p53-DNA binding at cruciform-forming target sequences. Interestingly, p53 interaction with Holliday junctions (another four-way junction motif occurring in DNA) has been reported [99] and it was suggested that p53 plays an important role in protecting this structure against endonuclease cleavage. The p53 protein has been described to exhibit a binding preference to cruciforms even in the absence of a target sequence, demonstrated using various techniques including direct visualization by atomic force microscopy [149,150]. The correlation between negative DNA supercoiling (facilitating local transitions from B-form DNA into a cruciform at the inverted repeats) and p53 binding enhancement suggests the importance of spatial DNA assembly for effective p53 binding [2,92].

#### 2.3.6. Influence of Epigenetic Changes to p53-DNA Interactions

It is well-known that many basic biological processes including gene expression depend on chromatin epigenetic states, such as DNA methylation [151] and histone modifications [152], and on noncoding RNA-mediated regulation [153]. Changes in these epigenetic features are seen in neurological diseases and cancer and become important in clinical medicine [154]. These epigenetic phenomena are important for p53-DNA binding as well for local DNA structure formation. p53 targets are typically associated with histone marks of transcriptionally active chromatin (H3K4me3 and H3K36me3) in normal cells and cancer cell lines typically have lower levels of DNA methylation [155]. It was experimentally validated that methylation changes alter p53 regulation [156]. Examination of genome-wide data sets shows three classes of p53 targets—near transcriptional start sites (TSS), promoter-distal enhancer elements with dynamic histone acetylation upon p53 binding, and within regions of inaccessible chromatin [157]. These data point to p53 acting as a pioneer transcription factor [158], similarly to the FoxA transcription factor [159]. The presence of local DNA structures are associated with an active genome when transcription and replications lead to increased superhelicity favor local DNA structure formation and dynamic changes in DNA supercoiling in vivo determine DNA accessibility for transcription [34]. These changes of superhelicity lead to cruciform extrusion [160]. The positive effect of p53 binding to supercoiled DNA has been demonstrated [85,86] and DNA supercoiling changes transcription in general [161,162]. Interestingly, loss of histone modifications (H3K4me3 and H3K9/14ac) around the TSS correlates with G-quadruplex prone sequences [163]. G-quadruplex ligands change epigenetic modification and, therefore, targeting of local DNA structures was suggested as a tool for specific epigenetic reprogramming [164]. Considering that cruciform structures as well for G-quadruplex have been shown to protect DNA from methylation [165,166], formation of local DNA structures could be an important additional factor in the p53 pioneering and DNA binding activities.

## 3. Conclusions

**Figure 6:**
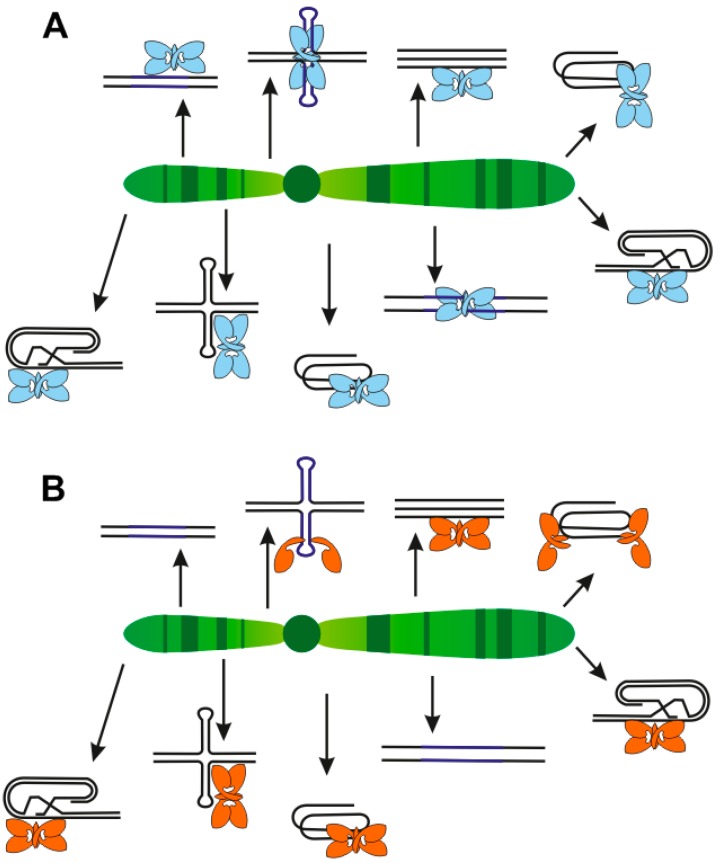
Various targets for wild-type (**A**-blue) and mutant (**B**-violet) proteins.

p53 binding to DNA is a basic feature important for its transcriptional-regulation function. In dependence of chromatin state, p53 shows the ability to bind to its target sequences in double-stranded DNA (reviewed in [2,46,47,48,49,50,51,52]) and/or to local non-B DNA structures [83] that may or may not be associated with a p53 consensus target sequence (Figure 6).

These local DNA structures represent a variety of DNA targets also as single-, three-, and four-stranded DNA. Interestingly, simultaneous binding of p53 to sequence- and structure-specific motifs shows remarkable cooperativity, evidenced by the proximity effects of triplex DNA located close to p53 double-stranded target DNA [37] and by the structural transition of symmetrical consensus target sequences from double-stranded B-DNA into corresponding cruciform structures [104] (Figure 4). These synergistic effects are observed not only at the level of increased protein-DNA binding affinity but also at the level of enhanced transcriptional activity. Some other local DNA structures, such as G-quadruplexes, have been identified as targets for mutant p53 proteins, which have lost sequence-specific DNA binding. In general, the C-terminal part of p53 is required for its effective structure-selective DNA binding. Moreover, tetrameric p53 binds DNA in a cooperative manner and activation of the apoptosis program is dependent on DNA binding cooperativity, while p53 mutants with reduced or increased cooperativity change the cell fate [167,168]. Mutant p53 with “gain of function” properties, which can be related to the mutant protein binding to certain structural motifs such as G-quadruplexes, plays a critical role in human tumor progression. It has been demonstrated that functional loss of p53 influences cell microenvironments and promotes malignant progression [169]. Mutant p53 also promotes invasiveness of cancer cells by extensive gene upregulation, for example, murine mutant p53R270H (the equivalent of human R273H) is associated with activation of inflammatory and innate immune pathways [170]. It was also demonstrated that dominant-negative and “gain of function” features of mutant p53 could be reduced by allele-specific siRNAs against p53 hotspot mutants [171]. While mutant p53 imbalanced p53-DNA binding equilibrium, targeted degradation of mutant p53 seems to be a promising strategy for treatment [172]. Moreover, the role of p53 isoforms has emerged in recent years [70,173,174]. Due to the altered C-terminal parts of p53β and p53γ isoforms, their DNA binding specificity to local DNA structures can be very different in comparison to the canonical p53α isoform. The evaluation of individual p53 mutants and p53 isoforms interacting with local DNA structures will shed light on the complex regulatory pathways controlled through orchestration of processes involving the p53 protein.
